# Additive anti-inflammatory effects of corticosteroids and phosphodiesterase-4 inhibitors in COPD CD8 cells

**DOI:** 10.1186/s12931-016-0325-8

**Published:** 2016-01-25

**Authors:** Seamus Grundy, Jonathan Plumb, Manminder Kaur, David Ray, Dave Singh

**Affiliations:** Centre for Respiratory Medicine and Allergy, Institute of Inflammation and Repair, Manchester Academic Health Science Centre, The University of Manchester and University Hospital of South Manchester, NHS Foundation Trust Southmoor Road, Manchester, M23 9LT UK; School of Medicine and Manchester Academic Health Science Centre, University of Manchester, Oxford Road, Manchester, M13 9PT UK

**Keywords:** Chronic Obstructive Pulmonary Disease, CD8, Corticosteroid, Phosphodiesterase 4 inhibitor

## Abstract

**Background:**

CD8 lymphocytes play an important role in the pathogenesis of COPD. Corticosteroids and phosphodiesterase 4 (PDE4) inhibitors are anti-inflammatory drugs used for COPD treatment. Little is known of the combined effect of these drugs on COPD CD8 cells. We studied the effect of corticosteroid combined with PDE4 inhibitors on cytokine release form circulating and pulmonary CD8 cells, and on glucocorticoid (GR) nuclear translocation.

**Methods:**

The effect of dexamethasone alone and in combination with the PDE4 inhibitors roflumilast and GSK256066 on cytokine release from circulating and pulmonary CD8 cells was measured. The effect of the compounds on nuclear translocation of GR and cyclic AMP-responsive element-binding protein (CREB) was studied using immunofluorescence.

**Results:**

Dexamethasone inhibited cytokine release from COPD CD8 cells in a concentration dependent manner. PDE4 inhibitors enhanced this anti-inflammatory effect in an additive manner. PDE4 inhibitors did not increase corticosteroid induced GR nuclear translocation. PDE4 inhibitors, but not corticosteroid, increased phospho-CREB nuclear translocation.

**Conclusion:**

The combination of corticosteroids and PDE4 inhibitors results in an additive anti-inflammatory effect in COPD CD8 cells. This enhanced anti-inflammatory effect could translate to important clinical benefits for patients with COPD.

**Electronic supplementary material:**

The online version of this article (doi:10.1186/s12931-016-0325-8) contains supplementary material, which is available to authorized users.

## Background

Chronic Obstructive Pulmonary Disease (COPD) is characterised by airflow obstruction and an abnormal inflammatory response to the inhalation of noxious particles, most commonly from cigarette smoking [1]. Lymphocytes play a key role in this inflammatory response; in particular, CD8 cell numbers are increased in the lungs of COPD patients [2–4]. These cells are capable of secreting pro-inflammatory cytokines and cytotoxic molecules such as perforin and granzymes that cause cell death [5, 6].

Inhaled corticosteroids (ICS) are widely used anti-inflammatory treatments for COPD. These drugs bind to the cytoplasmic glucocorticoid receptor (GR), forming a complex that translocates to the nucleus, thereby suppressing the activity of transcription factors such as nuclear factor kappa-light-chain-enhancer of activated B cells (NF-κB) that promote inflammatory gene transcription [7]. ICS are used primarily to prevent exacerbations in COPD patients with a history of exacerbations [8].

Phosphodiesterase 4 (PDE4) inhibitors are the only new class of anti-inflammatory therapy to be licensed for COPD in recent years. PDE4 inhibitors decrease degradation of cyclic adenosine monophosphate (cAMP) in immune cells, leading to a reduction in pro-inflammatory activity. Roflumilast is the only currently licensed PDE4 inhibitor, and is used to prevent exacerbations in severe COPD patients with a history of exacerbations and chronic bronchitis [9, 10]. This PDE4 inhibitor is an oral treatment that can have systemic side effects such as weight loss and gastro-intestinal disturbance. This had led to efforts to develop inhaled PDE4 inhibitors, such as GSK256066, in order to improve the therapeutic index [11].

Roflumilast and ICS target different cell signalling pathways, and it has been shown combining these drugs in vitro, using healthy human peripheral blood mononuclear cells (PBMCs) and COPD bronchial epithelial cells, results in an additive anti-inflammatory effect [12, 13]. These in vitro findings are mirrored by the results from the recently published REACT clinical trial, which showed a reduction in exacerbation rates when roflumilast was added to ICS (plus long acting bronchodilator) treatment in COPD patients who were frequent exacerbators [14].

We have further investigated the anti-inflammatory potential of combining corticosteroids and PDE4 inhibitors, by using COPD lymphocytes. We focused on CD8 cells, studying the effects of these drugs alone and in combination on lymphocyte cytokine production. We also evaluated whether PDE4 inhibition enhances GR nuclear translocation.

## Methods

### Subjects

COPD patients, smokers with normal lung function (S) and healthy non-smokers (HNS) were recruited to obtain blood CD8 cells and PBMCs. COPD was diagnosed in accordance with the GOLD strategy document [1]. A separate group of patients who were undergoing lung resection for known or suspected lung cancer were recruited to obtain lung tissue from which to isolate pulmonary CD8 cells. Table 1 shows the patient demographics. The studies performed were approved by the local research ethics committee (South Manchester Research Ethics Committee, reference: 03/SM/396). All subjects gave written informed consent.

**Table 1:** Subject demographics

	Peripheral blood CD8			Pulmonary CD8	
	COPD(17)	S(10)	HNS(7)	COPD(6)	S(4)
Age (yrs)	62.6 (45–72)	51.9 (38–75)	55.8 (44–69)	58.7 (43–73)	61.3 (50–68)
Male/Female	14/3	3/7*	2/5*	5/1	4/0
FEV_1_ (L)	1.5 (0.09)	3.0 (0.34)*	3.06 (0.35)*	1.75 (0.24)	3.11 (0.56)*
FEV1 % predicted	53.6 (3.5)	96.1 (3.83)*	99.6 (6.23)*	58.7 (9.7)	104.4 (11.8)*
FEV1: FVC (%)*	47.2 (2.8)	79.1 (1.89)*	79.1 (2.87)*	58.9 (8.4)	76.7 (8.9)*
Current Smoker	8	5	0*	3	4
Smoking History (pkyr)	42 (5.3)	17.6 (4.0)*	0*	42.4 (15.5)	36 (25.7)
ICS	10	0*	0*	2	0*

Data presented as mean (SD) or median (range). Data for all experiments are combined in this table. There were no significant differences between demographics of subsets of volunteers for individual experiments. PBMC samples were paired with peripheral blood CD8 cells. Therefore separate demographics for PBMCs not displayed. Statistically significant differences between COPD and other patient groups are indicated by *p < 0.05. COPD: chronic obstructive pulmonary disease; S: smoker with normal lung function; HNS: healthy non-smoker; FEV_1_: Forced expiratory volume in 1 s; FVC: forced vital capacity; ICS: inhaled corticosteroids

### Isolation of cells

PBMCs were isolated by Ficoll-Paque (GE Healthcare, Bucks, UK) density gradient. Circulating CD8 cells were isolated from PBMCs using positive selection CD8 microbeads (Miltenyi Biotec, Bisley, UK) in accordance with manufacturer’s instructions.

Pulmonary CD8 cells were isolated as previously described [15]. Briefly, lung tissue was homogenised in a blender. An enriched population of lymphocytes was obtained by Percoll (GE Healthcare) density gradient at percoll concentrations of 40 and 70 %. CD8 cells were positively selected from the enriched population of pulmonary lymphocytes using positive selection CD8 microbeads (Miltenyi Biotec).

### Cell culture

PBMCs and peripheral blood CD8 cells were seeded in triplicate in 96 well plates at 100,000 cells per well. Pulmonary CD8 cells were seeded at 50,000 cells per well. Cells were pre-treated for 1 h with dexamethasone (10^−6^ M – 10^−10^ M) alone or in combination with GSK256066 (Stratech, Newmarket, UK, Spain), roflumilast (Sigma-Aldrich, Poole, UK) or forskolin (Sigma-Aldrich). Cells were then stimulated for 24 h with anti-CD2/3/28 beads (Miltenyi Biotec). Supernatants were harvested and stored at −20 °C until ELISA was performed.

### Cytokine analysis

Supernatants were analysed by ELISA according to manufacturer’s instructions; IL-2 (R&D Systems DuoSet, R&D Systems, Abingdon, UK), IFNγ (Ready-Set-Go Kit, eBioscience, Herts, UK). Lower limits of detection were 15.6 pg/ml and 4 pg/ml for IL-2 and IFNγ respectively.

### Immunofluorescence

Cytospins were prepared, air dried and fixed in 4 % paraformaldehyde. Cells were labelled for Glucocorticoid receptor alpha (GR, thermoscientific PA1-516) or phospho-CREB (Cell signalling #9191 s). Nuclei were counterstained with 4′6-diamidino-2-phenylindole (DAPI).

Digital micrographs were obtained using a Nikon Eclipse 80i microscope equipped with a QImaging digital camera and ImagePro Plus 5.1 software (MediaCybernetics,

Marlow, UK). Quantification of the nuclear region was determined using the mean fluorescence intensity (MFI) acquired by ImagePro Plus 5.1 software. Background fluorescence levels were subtracted and nuclear MFI was normalised to basal levels in order to calculate relative changes.

### Statistics

Data was analysed in GraphPad Prism and statistics performed in GraphPad Instat (GraphPad Software, SanDiego, CA, USA; http://www.graphpad.com). Cytokine data were normally distributed. ANOVA was performed to compare drug effects; where ANOVA *p* < 0.05, pairwise comparisons were made using Bonferroni multiple comparisons test.

Concentration response curve fitting was performed using least squares nonlinear regression. EC_50_ and IC_50_ were calculated using Graphpad Prism from the fitted concentration response curves.

Interaction ratios were calculated from the ratio of observed efficacy (I_O_) to expected efficacy (I_E_). The expected efficacy was calculated using the Abbott formula:

$$ {\mathrm{I}}_{\mathrm{E}}=\mathrm{A}+\mathrm{B}-\left(\mathrm{AB}/100\right) $$

when A is efficacy of compound A and B is efficacy of compound B.

An interaction ratio between 0.5 and 1.5 is consistent with an additive effect [16].

## Results

### Anti-inflammatory effects on blood lymphocytes

The concentration response effect of GSK256066, roflumilast and forskolin on IL-2 release from PBMCs was studied in order to choose concentrations for further experiments (Additional file 1 and Additional file 2: Figure S1). The following concentrations were selected for further experiments; GSK256066: 10^−9^M; roflumilast: 10^−7^M; forskolin: 10^−6^M. These concentrations were selected as they were below the maximum effect concentration (Emax) but were around the IC_50_ value thus ensuring pharmacological activity.

PBMCs and circulating CD8 cells were isolated from COPD patients (*n* = 13), S (*n* = 8) and HNS (*n* = 7). Basal levels of IL-2 and IFNγ were low for all groups. Anti-CD2/3/28 beads significantly increased the production of both IL-2 and IFNγ from CD8 cells and PBMCs (Additional file 1: Table S1). There were no significant differences between subject groups in levels of cytokine release from CD8 cells. In PBMCs, levels of stimulated IL-2 release were higher in S (mean 6414.6 pg/ml) and HNS (mean 6327.2 pg/ml) compared to COPD (mean 2469.3 pg/ml), p ≤ 0.05 for both comparisons.

**Fig. 1:**
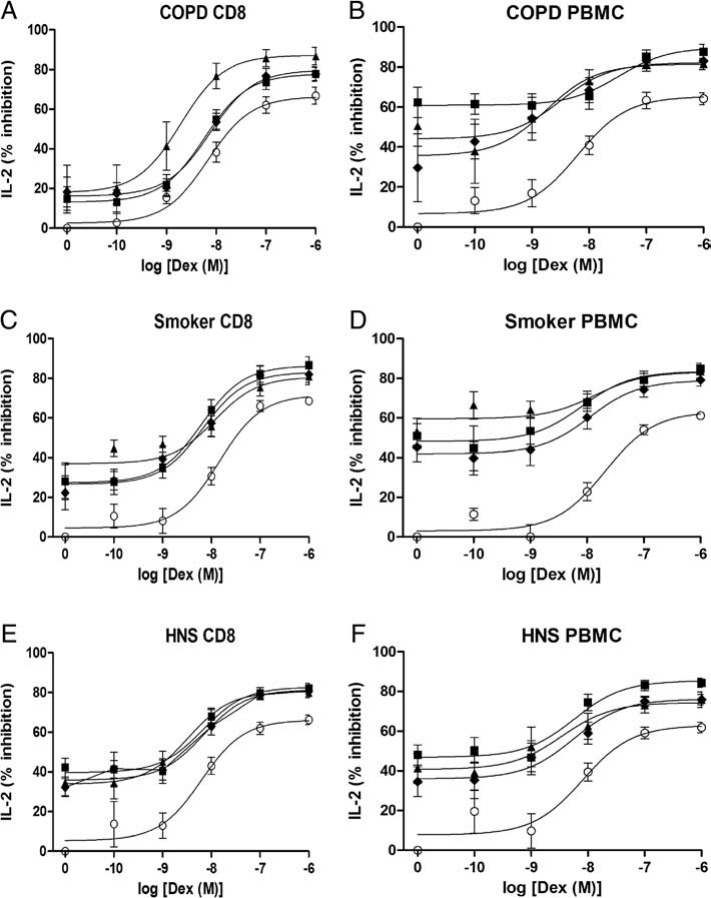
The effect of PDE4 inhibitors with dexamethasone on IL-2 release from peripheral blood CD8 cells and PBMCs. Peripheral blood CD8 cells and PBMCs from COPD (*n* = 13, **a** & **b**), Smoker (*n* = 8, **c** & **d**) and Healthy non-smokers (HNS, *n* = 7 **e** & **f**) were pre-treated for 1 h with various concentrations of dexamethasone (Dex) alone (О) or in combination with GSK256066 (■), Roflumilast (♦) or Forskolin (▲). The effect of GSK256066, roflumilast and forskolin alone is represented as log[Dex] = 0. Cells were stimulated for 24 h with anti-CD2/3/28 beads. Supernatants were harvested and Interleukin 2 (IL-2) was measured by ELISA. Data presented as mean ± SE

**Fig. 2:**
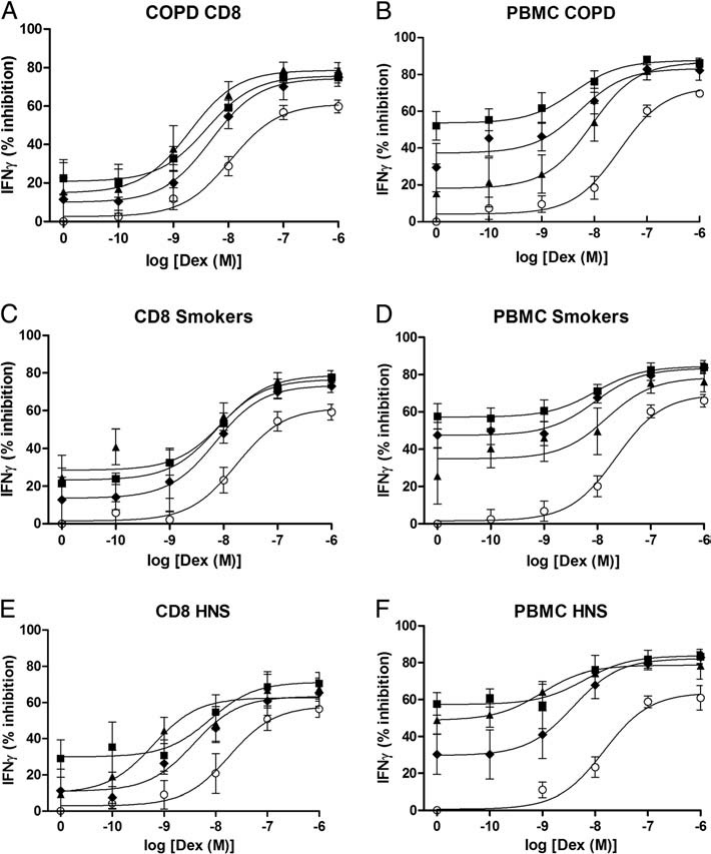
The effect of PDE4 inhibitors with dexamethasone on IFNγ release from peripheral blood CD8 cells and PBMCs. Peripheral blood CD8 cells and PBMCs from COPD (*n* = 13, **a** & **b**), Smoker (*n* = 8 **c** & **d**) and Healthy non-smokers (HNS,*n* = 7, **e** & **f**) were pre-treated for 1 h with various concentrations of dexamethasone (Dex) alone (О) or in combination with GSK256066 (■), Roflumilast (♦) or Forskolin (▲). The effect of GSK256066, roflumilast and forskolin alone is represented as log[Dex]M = 0. Cells were stimulated for 24 h with anti-CD2/3/28 beads. Supernatants were harvested and Interferon gamma (IFNγ) was measured by ELISA. Data presented as mean ± SE

Dexamethasone inhibited both IL-2 and IFNγ release from peripheral blood CD8 cells and PBMCs in a concentration dependent manner (Figs. 1 and 2). Inhibition at the highest dexamethasone concentration (10^−6^ M) ranged from 61.1 to 68.5 % for IL-2 and from 56.3 to 59.7 % for IFNγ in the different subject groups (Emax values are shown in Table 2). The inhibitory effect of dexamethasone was not statistically different between subject groups (ANOVA *p* > 0.05 for all concentrations of dexamethasone). Table 2 shows that the IC_50_, EC_50_ and Emax values were numerically similar between groups.

**Table 2:** Mean EC_50_, IC_50_ and E_max_ for dexamethasone alone and in combination with PDE4 inhibitors in blood CD8 cells and PBMCs

		Dex	Dex/256066	Dex/Rof	Dex/Forsk
		Mean EC_50_ IL-2			
CD8	COPD	7.8	4.8	6.7	1.6
	Smoker	16.6	5.4	6.1	14.1
	HNS	11.2	5.4	7.7	2.7
PBMC	COPD	8.91	11.4	4.2	2.2
	Smoker	22.9	8.3	8.7	18.2
	HNS	12.0	4.9	3.8	2.0
		Mean EC_50_ IFNγ			
CD8	COPD	10.0	4.0	5.0	1.6
	Smoker	20.0	6.3	7.9	10.0
	HNS	15.8	12.6	3.2	5.0
PBMC	COPD	31.6	4.0	6.3	10.0
	Smoker	20.0	10.0	7.9	15.8
	HNS	10.0	4.0	2.5	2.5
		Mean IC_50_ IL-2			
CD8	COPD	7.9	5.5	7.1	1.8
	Smoker	16.6	6.2	8.5	22.9
	HNS	7.9	5.9	8.3	3.2
PBMC	COPD	8.5	30.2	4.8	1.6
	Smoker	22.9	6.6	9.3	33.9
	HNS	10.7	5.5	6.5	3.1
		Mean IC_50_ IFNγ			
CD8	COPD	12.0	3.6	4.6	1.9
	Smoker	18.2	8.1	7.4	11.0
	HNS	21.4	8.1	3.6	0.8
PBMC	COPD	33.1	5.0	8.5	9.8
	Smoker	23.4	8.7	8.9	24.0
	HNS	15.1	6.0	3.6	1.0
		E_max_ IL2			
CD8	COPD	67.9	78.1	78.2	86.6
	Smoker	68.5	86.5	81.9	80.9
	HNS	66.3	82.0	80.7	79.7
PBMC	COPD	64.2	87.4	83.0	81.6
	Smoker	61.1	84.8	79.2	84.1
	HNS	61.9	84.3	75.9	77.5
		E_max_ IFNγ			
CD8	COPD	59.8	74.9	75.1	78.8
	Smoker	59.2	77.6	72.9	76.6
	HNS	56.3	70.5	65.4	75.4
PBMC	COPD	69.7	86.4	82.2	85.7
	Smoker	66.0	84.0	83.6	75.4
	HNS	61.0	83.2	82.9	73.7

Data presented mean nanomolar concentration for EC_50_ and IC_50_. Data presented as mean percent inhibition for E_max_. Dex: dexamethasone; 256066: GSK256066;CD8: circulating CD8 cells; PBMC: peripheral blood mononuclear cells; Rof: roflumilast; Forsk: forskolin; COPD: chronic obstructive pulmonary disease; HNS: healthy non-smoker; IL-2: interleukin 2; IFNγ: interferon gamma; EC_50_:concentration producing 50 % of maximal effect; IC_50_: concentration producing reduction of 50 % relative to untreated stimulated levels; E_max_: Maximal inhibitory effect at dexamethasone 10^−6^ M

GSK256066 (10^−9^ M), roflumilast (10^−7^ M) and forskolin (10^−6^ M) each caused significant inhibition of both IL-2 and IFNγ release from stimulated peripheral blood CD8 cells and PBMCs. The inhibitory effects of these compounds was generally <60 % (Figs. 1 and 2; data points on graphs where log[Dex] = 0).

Dexamethasone combined with GSK256066, roflumilast or forskolin resulted in greater inhibition of cytokine release in all subject groups (Figs. 1 and 2, Table 2, Additional file 1: Table S2). This enhanced inhibition was significantly greater than the effect of corticosteroid alone (ANOVA *p* < 0.05, Bonferroni *p* < 0.05) for all conditions except for the dexamethasone 10^−10^ M concentration for both IL-2 and IFNγ from CD8 cells.

The EC_50_ and IC_50_ values were generally lower for dexamethasone combined with GSK256066, roflumilast or forskolin compared to dexamethasone alone in all subject groups (Table 2). Similarly, the Emax values for combination treatments were greater than dexamethasone alone. Calculated interaction ratios were consistent with an additive effect (for example at dexamethasone 10^−8^ M the majority of IR values ranged from 1.0-1.5 for both PBMCs and CD8 cells; see Additional file 1: Table S2).

### Anti-inflammatory effects on pulmonary CD8 cells

Pulmonary CD8 cells were isolated from lung tissue of COPD patients (*n* = 6) and S (*n* = 4). Due to limited cell numbers these experiments were limited to a single PDE4 inhibitor. We selected GSK256066 as it was the most potent in the previous experiments (See Additional file 1: EC_50_ data). Basal levels of IL-2 and IFNγ release from pulmonary CD8 cells were low. Anti-CD2/3/28 beads induced a significant increase in release of both IL-2 and IFNγ from pulmonary CD8 cells (Additional file 1: Table S1). There were no significant differences between patient groups in levels of cytokine release from pulmonary CD8 cells (*p* > 0.05 for both IL-2 and IFNγ).

**Fig. 3:**
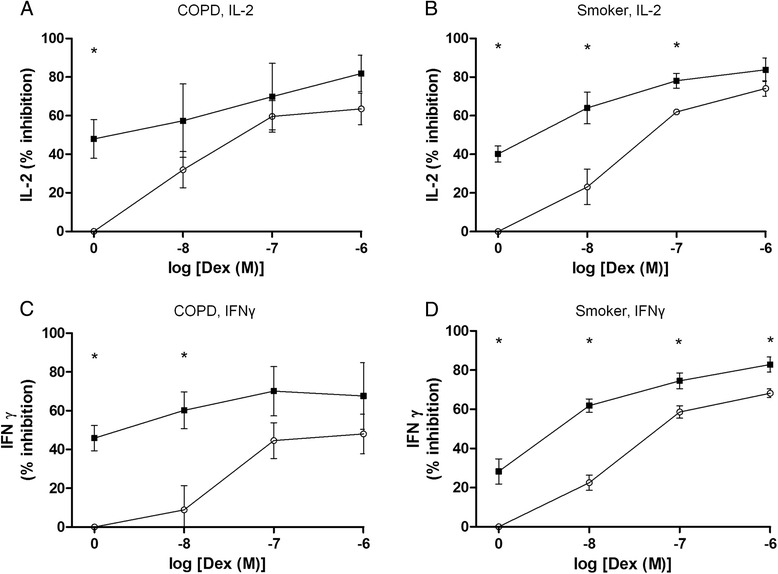
The effect of GSK256066 with dexamethasone on cytokine release from pulmonary CD8 cells. Pulmonary CD8 cells from COPD (*n* = 6) and smokers with normal lung function (*n* = 4) were pre-treated with various concentrations of dexamethasone (Dex) alone (О) or in combination with GSK256066 (■). The effect of GSK256066 alone is represented as log[Dex]M = 0. Cells were then stimulated with anti-CD2/3/28 beads for 24 h. Supernatants were harvested and interleukin 2 (IL-2) and interferon gamma (IFNγ) were measured by ELISA. Data presented as mean ± 95 % confidence intervals. **a** COPD, IL-2, **b** Smokers, IL-2, **c** COPD, IFNγ, **d** Smoker. IFNγ. Statistical significance of difference between dexamethasone alone compared to dexamethasone with GSK256066 indicated by * *p* < 0.05

GSK256066 significantly inhibited release of both IL-2 (mean percent inhibition 40.1 % in S, 47.9 % in COPD) and IFNγ (mean percent inhibition 28.5 % in S, 45.8 % in COPD) from pulmonary CD8 cells with no significant differences between patient groups (Fig. 3). Dexamethasone inhibited cytokine release from pulmonary CD8 cells in a concentration dependent manner with mean inhibition at the highest concentration for IL-2 of 74.1 and 63.5 % for S and COPD respectively and IFNγ of 68.2 and 48.0 % for S and COPD respectively (Fig. 3). There were no significant differences in effect of dexamethasone between patient groups.

Dexamethasone combined with GSK256066 produced a greater anti-inflammatory effect than dexamethasone alone in both subject groups (Fig. 3). This enhanced anti-inflammatory effect was numerically apparent at most concentrations, but did not always achieve statistical significance. Interaction ratios ranged from 0.9 to 1.4 for all studied concentrations of dexamethasone (Table 3). This is consistent with an additive effect for dexamethasone and GSK256066 combined.

**Table 3:** Interaction ratios for combination of dexamethasone and GSK256066 on pulmonary CD8 cells

	COPD			Smokers		
	Interaction ratio IL-2					
Dex (M)	10^−8^	10^−7^	10^−6^	10^−8^	10^−7^	10^−6^
I_o_	57.3	69.9	81.1	64.0	78.1	83.8
I_E_	64.5	79.0	81.0	54.1	77.2	84.6
IR	0.9	0.9	1.0	1.2	1.1	1.0
	Interaction ratio IFNγ					
Dex (M)	10^−8^	10^−7^	10^−6^	10^−8^	10^−7^	10^−6^
I_o_	60.2	70.1	67.6	61.9	74.6	82.9
I_E_	50.7	70	71.9	44.4	70.4	77.2
IR	1.2	1.0	0.9	1.4	1.1	1.1

Data presented as percent inhibition relative to cells stimulated with anti-CD2/3/28 I_O_ & I_E_. IR is the ratio of I_O_ to I_E._ COPD: chronic obstructive pulmonary disease; Dex: dexamethasone; IL-2: interleukin 2; IFNγ: interferon gamma; I_O_: Observed inhibition; I_E_: Expected inhibition; IR: interaction ratio. An interaction ratio between 0.5 and 1.5 is consistent with additive effect

### GR nuclear translocation

**Fig. 4:**
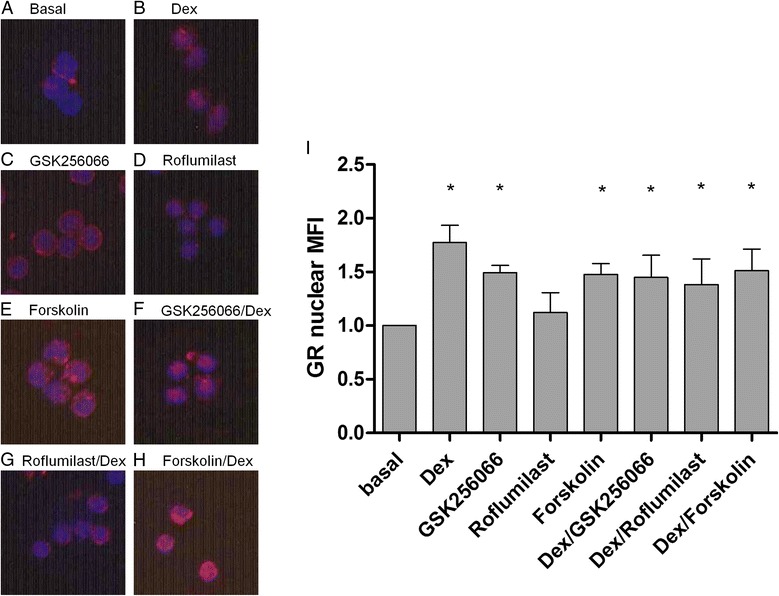
Effect of Dexamethasone and PDE4 inhibitors on nuclear translocation of Glucocorticoid Receptor. Peripheral blood CD8 cells from COPD patients (*n* = 5) were untreated (basal), treated with GSK256066 (10^−9^ M), roflumilast (10^−7^ M), or forskolin (10^−6^ M) alone or treated with dexamethasone (10^−6^ M, Dex) alone and in combination with GSK256066, roflumilast or forskolin. Cells were harvested after 60 min. Cells were immunostained with rabbit anti-glucocorticoid receptor (GR) antibody (red). Nuclei were counterstained with 4′,6-diamidino-2-phenylindole (blue). The nuclear mean fluorescence intensity (MFI) of GR was measured. Representative images of each experimental treatment are shown (**a**-**g**). **h** Data is presented as mean ± SE GR nuclear MFI normalised to basal levels. Statistically significant increase in nuclear GR above basal is indicated when *p < 0.05

In resting blood CD8 cells from COPD patients (*n* = 5), GR was predominantly visualised within the cytoplasm. Treatment with dexamethasone resulted in nuclear translocation of GR. Treatment with GSK256066 and forskolin alone also produced nuclear translocation of GR. Roflumilast alone had no effect on the sub-cellular location of GR. The combination of dexamethasone with GSK256066, roflumilast or forskolin did not result in any greater level of nuclear GR compared to dexamethasone alone (Fig. 4).

### CREB nuclear translocation

**Fig. 5:**
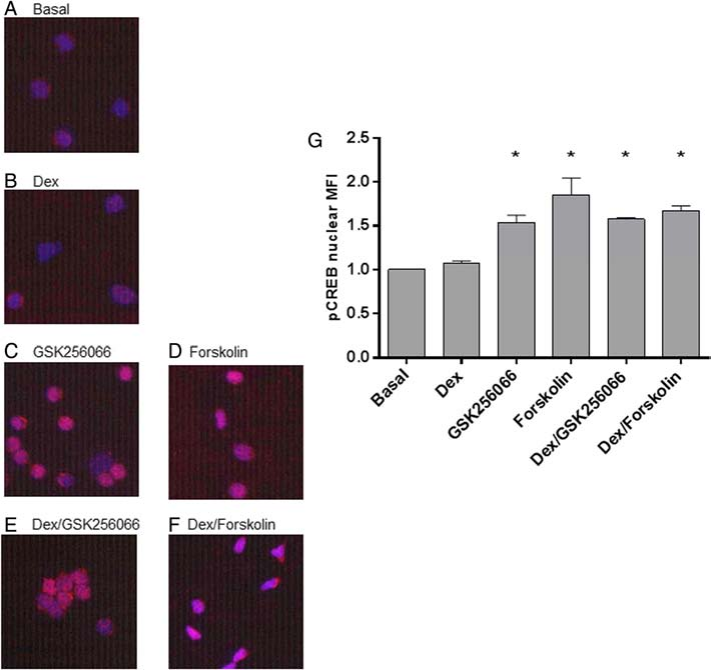
The effect of dexamethasone and PDE4 inhibitors of nuclear translocation of phoshporylated-CREB. Peripheral blood CD8 cells were isolated from 4 COPD patients. Cells were treated with GSK256066 (10^−9^ M) or forskolin (10^−6^ M) alone or dexamethasone (10^−6^ M) alone and in combination with GSK256066 or forskolin. Cells were immunostained with rabbit anti-phospho-cAMP response element binding (pCREB, red). Nuclei were counterstained with 4′,6-diamidino-2-phenylindole (blue). The nuclear mean fluorescence intensity (MFI) of pCREB was measured. Representative images of each experimental condition (**a**-**g**). Data is presented as mean ± SE pCREB nuclear MFI (H). Statistically significant increase in nuclear pCREB is indicated when * *p* < 0.05

In unstimulated peripheral blood CD8 cells, dexamethasone had no significant effect on nuclear translocation of p-CREB. In contrast, GSK256066 and forskolin increased nuclear p-CREB localisation. The addition of dexamethasone to GSK256066 or forskolin had no additional effect on p-CREB nuclear translocation compared to GSK256066 alone (Fig. 5).

## Discussion

The combination of dexamethasone with PDE4 inhibitors had an additive anti-inflammatory effect on both circulating and pulmonary CD8 cells from COPD patients and controls. These drug classes can be used in combination in clinical practice [14]; we show that this clinical strategy causes additive anti-inflammatory effects on a cell type involved in COPD inflammation. This additive anti-inflammatory effect is due to PDE4 inhibitors influencing cAMP signalling, which is not affected by corticosteroids.

We used sub-maximal concentrations of the PDE4 inhibitors roflumilast and GSK256066 to evaluate interactions with corticosteroid. In PBMCs, which contain both CD4 and CD8 cells, and isolated CD8 cells, we observed the same phenomenon; PDE4 inhibition provided an additional anti-inflammatory effect on top of that caused by dexamethasone. This resulted in a greater Emax (maximal inhibitory effect) and a reduced EC_50_ value for combination treatment. It is interesting that this additive effect was present at the majority of corticosteroid concentrations, as it implies that this effect may also be observed in clinical practice across a range of drug concentrations. The use of forskolin, which is known to increase cAMP levels, in these experiments provided confirmation that the additive effect was due to the combination of cAMP modulation with GR activation. Previous work has suggested that cAMP signalling may oppose glucocorticoid action [17, 18]. However, we show that this does not occur in COPD lymphocytes, as an additive effect is observed with simultaneous cAMP modulation with GR activation.

The number of experiments possible with lung cells was reduced due to the lower number of cells available. We chose to only use GSK256066 for these experiments, as it had been the most potent in our hands, which is consistent with previous publications comparing this drug to roflumilast [19]. The results from lung CD8 cells showed the same pattern as blood, but the experimental variability was greater and so statistical significance was not reached for every concentration when analysing whether two drugs had a greater effect than one. Nevertheless, our results overall show the potential for additive anti-inflammatory effects on COPD CD8 cells when PDE4 inhibitors and corticosteroids are used together.

The calculation of the IR indicated additive, and not synergistic, effects on cytokine production. Furthermore, we saw no enhancement of ligand dependent GR nuclear translocation by PDE4 inhibition. Previous studies in lung cell lines, such as epithelium and airway smooth muscle, have shown a synergistic effect when corticosteroids are combined with compounds that increase intra-cellular cAMP levels [20–22]. We did not observe synergy in lymphocytes, indicating that synergy between PDE4 inhibitors and corticosteroids does not occur in all cell types. Similarly, Toll-like receptor 3 stimulation of COPD bronchial epithelial cells resulted in cytokine production that was suppressed by PDE4 inhibition and corticosteroid in an additive, and not synergistic, manner [12]. This further supports the concept that additive or synergistic interactions between these drugs classes vary according to the cell type used, and whether these are cell lines or freshly obtained primary cells from COPD patients.

Ligand-independent nuclear translocation of GR is known to occur in response to β_2_-adrenoreceptor agonists [23]. Similar to PDE4 inhibitors, β_2_-adrenoreceptor agonists also increase intracellular cAMP levels. We also observed some evidence that PDE4 inhibition and forskolin (without corticosteroid) enhance GR nuclear translocation. Thus, it seems likely that cAMP has a direct effect on GR nuclear translocation in lymphocytes. However, GR nuclear translocation caused by cAMP may not result in enhanced GR anti-inflammatory activity, and it would be necessary to study DNA binding of GR in response to PDE4 inhibitors to understand this. Furthermore, there was no additive GR nuclear translocation when PDE4 inhibitors were used with corticosteroids in lymphocytes, indicating that this is not a mechanism that causes additive anti-inflammatory effects.

PDE4 inhibitors, but not corticosteroid, increased nuclear p-CREB; this transcription factor is known to be cAMP responsive. This observation provides evidence that PDE4 inhibition modulates cell signalling pathways that are unaffected by corticosteroids. cAMP is known to regulate production of cytokines such as IL-2 [24, 25], while corticosteroids suppress pro-inflammatory cytokine transcription through transrepression mechanisms such as interference with NF-κB activity [7]. The targeting of different signalling pathways offers the likely explanation for additive effects reported here when these drug classes are combined. Beta agonists modulate cAMP signalling, and there is evidence that roflumilast interacts with beta-agonists in epithelial cells to enhance corticosteroid effects. This is worthy of study in COPD lymphocytes.

The anti-inflammatory effects of dexamethasone were similar in COPD patients and controls, both in PBMCs and CD8 cells from the blood and lung tissue. We have previously reported similar corticosteroid sensitivity in COPD and control CD8 cells from blood and lung tissue [15]. In contrast, stimulated bronchoalveolar lavage lymphocytes, containing CD4 and CD8 cells, show decreased corticosteroid sensitivity in COPD patients compared to controls [26]. This highlights the potential of corticosteroid effects on COPD lymphocytes to differ according to the anatomical location of the cells, and possibly the ratio of CD4 and CD8 cells present.

CD8 cells are capable of playing a variety of pro-inflammatory roles in COPD, such as the secretion of pro-inflammatory cytokines and cytotoxic molecules including perforin and granzymes [5, 6]. These cells are commonly involved in the host response to pathogens such as viruses and bacteria. COPD exacerbations are often caused by viruses and bacteria, and are characterised by an excessive pro-inflammatory response to the pathogen [27]. The role of cytotoxic CD8 cells in the response to virus infection is well known [28], and these cells also produce a pro-inflammatory response to bacteria; this has been demonstrated using COPD lung CD8 cells [29]. Inhaled corticosteroids are used to prevent the excessive immune response that occurs during exacerbations. The pharmacological interaction demonstrated in this paper may be useful for limiting any role that CD8 cells play in the inflammatory cascade during COPD exacerbations.

We used T-cell receptor stimulation to evaluate the effects of drugs on CD8 cytokine production. CD8 cells express TLR receptors [30], and it would be interesting to investigate the effects of corticosteroids and PDE4 inhibitors on TLR stimulated CD8 cells. This could include a wider range of cytokines than those studied here, such as IL-17, IL-13 and IL-10.

Stimulated IL-2 levels from PBMCs were lower in COPD patients compared to controls. This pattern was not observed for IFNγ secretion from PBMCs, nor were there any differences between COPD patients and controls for cytokine release from blood or lung CD8 cells. The lower IL-2 levels from COPD PBMCs may be a false positive finding in a relatively limited sample size, as there was no other evidence for reduced cytokine production in our other experiments. Other papers investigating lymphocyte cytokine secretion from PBMCs have produced varying results, with more evidence for increased secretion from COPD cells [31–34].

## Conclusions

Clinical data shows that roflumilast causes additional clinical benefits when added to ICS in COPD patients [14, 35]. Our COPD lymphocyte data indicates that the combination of these drugs causes an additive anti-inflammatory effect on lymphocyte cytokine production. Given the important role of lymphocytes in the pathophysiology of COPD, our results could represent one mechanism by which the clinical benefit of combining these drug classes occurs. Furthermore, our data support combining these drug classes in order to increase the magnitude of anti-inflammatory effect on COPD lung cells.

## Additional files

Additional file 1: Supplementary data and Tables S1 & S2. (DOCX 20 kb) Additional file 2: Figure S1. Effect of GSK256066, Roflumilast and Forskolin on release of IL-2 in peripheral blood CD8 cells. Peripheral blood CD8 cells from healthy non-smokers (*n* = 2) were pre-treated with stated concentrations of GSK256066 (A), Roflumilast (B) or Forskolin (C) for 1 h prior to stimulation with anti-CD2/3/28 beads for 24 h. Supernatants were harvested and interleukin 2 (IL-2) was measured by ELISA. Data presented as mean ± SE % inhibition of IL-2. (PNG 43 kb)
