# Supplementary material for: Additive anti-inflammatory effects of corticosteroids and phosphodiesterase-4 inhibitors in COPD CD8 cells
Source: Respir Res. 2016 Jan 25;17:9. doi: 10.1186/s12931-016-0325-8 (PMC4727404; doi:10.1186/s12931-016-0325-8)
Supplement: Additional file 1: — Supplementary data and Tables S1 & S2. (DOCX 20 kb) [file 12931_2016_325_MOESM1_ESM.docx]

**Dose Response Curves**

The concentration response effects of GSK256066, roflumilast and forskolin on IL-2 production from PBMCs from 2 healthy non-smokers was investigated in order to select concentrations with submaximal effects for further experiments. Basal levels of IL-2 were undetectable. Stimulation with anti-CD2/3/28 beads resulted in significant IL-2 release (mean 1874.1 pg/ml). Sigmoidal concentration responses for all three compounds were observed (figure 1). The EC_50_ for GSK256066, roflumilast and forskolin were 0.06nM, 29.4nM and 600nM respectively. The maximum % inhibition of IL-2 at a concentration of 10^-6^M was 53.8%, 74.4% and 60.2% for roflumilast, GSK256066 and forskolin respectively. The following concentrations were selected for further experiments: GSK256066 (10^-9^M), roflumilast (10^-7^M), forskolin(10^-6^M).

|  | **PBMC** | | | **Circulating**  **CD8** | | | **Pulmonary CD8** | |
| --- | --- | --- | --- | --- | --- | --- | --- | --- |
|  | **HNS** | **S** | **COPD** | **HNS** | **S** | **COPD** | **S** | **COPD** |
| **Basal IL-2** | 39.9 (105.5) | 0  (0) | 3.3  (8) | 0  (0) | 7.4  (21) | 0  (0) | 2.2  (4) | 0  (0) |
| **Stimulated IL-2** | 6327.2 (2271) | 6414.6 (1621) | 2469.3 (1407) | 4680.9 (3820) | 5639.0 (4567) | 3913.0 (4446) | 751.3 (419) | 853.0 (807) |
| **Basal IFNγ** | 151.4 (262) | 66.5 (108) | 162.8 (270) | 26.0 (30) | 54.3 (72) | 25.9 (40) | 28.2 (47) | 22.8 (20) |
| **Stimulated IFNγ** | 7382.8 (6025) | 7219.3 (8305) | 11537.8 (9512) | 1885.5 (1659) | 2797.4 (2356) | 1484.9 (264) | 2666.9 (3210) | 3392.2 (2513) |

**Supplementary Table 1. Basal and Stimulated levels of IL-2 and IFNγ.**

Data are presented as mean (SD) pg/ml. Cells were stimulated using anti-CD2/3/28 beads at a cell to bead ration of 0.5:1. PBMC: peripheral blood mononuclear cells; HNS: healthy non-smoker; S: Smoker; COPD: Chronic obstructive pulmonary disease; IL-2: interleukin 2; IFNγ: interferon gamma.

**Supplementary table 2. Interaction ratios for dexamethasone combined with PDE4 inhibitors in circulating CD8 cells and PBMCs.**

|  | **COPD** | | | **Smokers** | | | **HNS** | | |
| --- | --- | --- | --- | --- | --- | --- | --- | --- | --- |
|  | **IL-2 CD8 cells** | | | | | | | | |
|  | **256066** | **Rof** | **Forsk** | **256066** | **Rof** | **Forsk** | **256066** | **Rof** | **Forsk** |
| **I_O_** | 54.8 | 53.6 | 76.7 | 63.9 | 57.7 | 55.8 | 67.9 | 62.9 | 68.4 |
| **I_E_** | 47.4 | 49.4 | 50.3 | 50.1 | 46.0 | 50.1 | 67.0 | 61.4 | 65.6 |
| **IR** | 1.2 | 1.1 | 1.5 | 1.3 | 1.3 | 1.1 | 1.0 | 1.0 | 1.0 |
|  | **IFNγ CD8 cells** | | | | | | | | |
| **I_O_** | 59.2 | 54.5 | 65.7 | 53.2 | 47.9 | 56.9 | 54.6 | 45.9 | 48.2 |
| **I_E_** | 44.7 | 37.1 | 39.9 | 39.6 | 33.0 | 42.0 | 43.7 | 29.7 | 28.1 |
| **IR** | 1.3 | 1.5 | 1.6 | 1.3 | 1.4 | 1.4 | 1.2 | 1.5 | 1.7 |
|  | **IL-2 PBMCs** | | | | | | | | |
| **I_O_** | 65.4 | 68.4 | 73.1 | 67.7 | 60.1 | 69.1 | 74.5 | 59.1 | 62.5 |
| **I_E_** | 77.7 | 58.4 | 70.7 | 62.1 | 57.7 | 63.8 | 68.5 | 60.3 | 64.4 |
| **IR** | 0.8 | 1.2 | 1.0 | 1.1 | 1.0 | 1.1 | 1.1 | 1.0 | 1.0 |
|  | **IFNγ PBMCs** | | | | | | | | |
| **I_O_** | 72.2 | 66.0 | 54.1 | 71.1 | 67.6 | 49.6 | 76.0 | 67.9 | 74.3 |
| **I_E_** | 60.9 | 42.5 | 31.1 | 66.2 | 58.2 | 40.6 | 67.5 | 46.5 | 60.8 |
| **IR** | 1.2 | 1.5 | 1.7 | 1.1 | 1.2 | 1.2 | 1.1 | 1.5 | 1.2 |

Data for I_O_ and I_E_ presented as percent inhibition produced by dex 10^-8^ M relative to cells stimulated with anti-CD2/3/28 . IR is the ratio of I_O_ to I_E._ COPD: chronic obstructive pulmonary disease; Dex: dexamethasone; IL-2: interleukin 2; IFNγ: interferon gamma; I_O_:Observed inhibition; I_E_: Expected inhibition; IR: interaction ratio. An interaction ratio between 0.5 and 1.5 is consistent with additive effect.
