# Supplementary figures and images for: Additive anti-inflammatory effects of corticosteroids and phosphodiesterase-4 inhibitors in COPD CD8 cells
Source: Respir Res. 2016 Jan 25;17:9. doi: 10.1186/s12931-016-0325-8 (PMC4727404; doi:10.1186/s12931-016-0325-8)

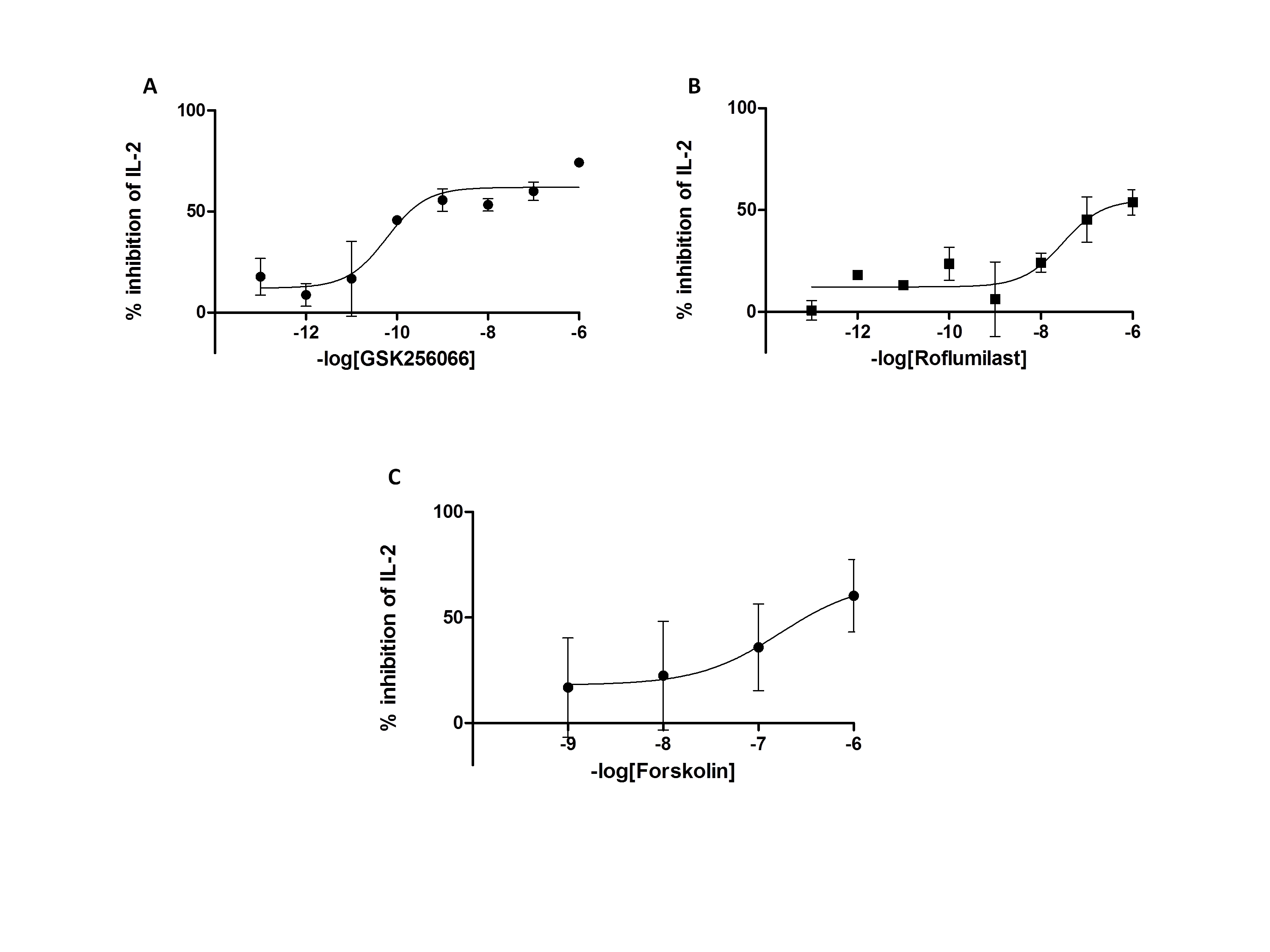

Supplement: Additional file 2: Figure S1. — Effect of GSK256066, Roflumilast and Forskolin on release of IL-2 in peripheral blood CD8 cells. Peripheral blood CD8 cells from healthy non-smokers (n = 2) were pre-treated with stated concentrations of GSK256066 (A), Roflumilast (B) or Forskolin (C) for 1 h prior to stimulation with anti-CD2/3/28 beads for 24 h. Supernatants were harvested and interleukin 2 (IL-2) was measured by ELISA. Data presented as mean ± SE % inhibition of IL-2. (PNG 43 kb) [file 12931_2016_325_MOESM2_ESM.png]
